# Prophages: an integral but understudied component of the human microbiome

**DOI:** 10.1099/mgen.0.001166

**Published:** 2024-01-24

**Authors:** Laura K. Inglis, Michael J. Roach, Robert A. Edwards

**Affiliations:** ^1^​ Flinders Accelerator for Microbiome Exploration, College of Science and Engineering, Flinders University, Bedford Park, SA 5042, Australia

**Keywords:** phage, prophage, human microbiome, disease

## Abstract

Phages integrated into a bacterial genome – called prophages – continuously monitor the vigour of the host bacteria to determine when to escape the genome and to protect their host from other phage infections, and they may provide genes that promote bacterial growth. Prophages are essential to almost all microbiomes, including the human microbiome. However, most human microbiome studies have focused on bacteria, ignoring free and integrated phages, so we know little about how these prophages affect the human microbiome. To address this gap in our knowledge, we compared the prophages identified in 14 987 bacterial genomes isolated from human body sites to characterize prophage DNA in the human microbiome. Here, we show that prophage DNA is ubiquitous, comprising on average 1–5 % of each bacterial genome. The prophage content per genome varies with the isolation site on the human body, the health of the human and whether the disease was symptomatic. The presence of prophages promotes bacterial growth and sculpts the microbiome. However, the disparities caused by prophages vary throughout the body.

## Abbreviation

GI, gastrointestinal.

## Data Summary

The datasets created/analysed in this study are available at: NCBI Genome Assemblies Summary Archive 20220601 [1, 2], https://doi.org/10.25451/flinders.22299664.v2; Prophage predictions [1, 3], https://doi.org/10.25451/flinders.c.6629843 ; Archive of the PATRIC Metadata from 20220601 [1, 4], https://doi.org/10.25451/flinders.22299655.v2 ; Prophages in humans (this paper), https://doi.org/10.25451/flinders.24564379.v3

### Impact Statement

This paper searches an immense amount of human microbiome samples to find phages integrated into bacterial genomes, called prophages, an aspect of the microbiome that has been comparatively understudied. This paper draws comparisons between prophages in different areas of the human body, and prophage abundance and human health. It is the first study of prophages in the human microbiome with such a large dataset and will be a useful foundation for future work.

## Introduction

The human microbiome is a complex ecosystem of microbes that inhabit every part of the human body. Most body sites typically contain a multitude of microbes resulting in diverse ecosystems. In contrast, sites in the human body dominated by one or a few species – dysbiosis – are often an indicator of disease [5–7].

While the term ‘human microbiome’ may evoke the mental image of the human body as a single environment, the body contains many different niches. Environments such as the skin, stomach, lungs and mouth are so different from each other that, combined, they have an extensive range of bacterial concentrations, very high species richness and widely varying species diversities. Most of the total microbial biomass in humans and other mammals resides in the gut, and that organ’s metabolism contributes to the animal’s overall thermogenic energy expenditure [8]. Other body areas have orders of magnitude lower bacterial concentrations than the gut [9]. The gut microbiome is also highly diverse [10], while others, such as the lung microbiome, are dominated by only a few groups [11].

Bacteriophages (phages) are viruses that infect bacteria found in almost every environment [12]. In the ocean, they kill approximately 20 % of the microbial biomass daily [13], but their role in sculpting and controlling most microbiomes, including the human microbiome, is not fully understood. A few clades dominate gut phages, but phages infect almost all bacteria in the human gut [14, 15]. There are two main kinds of phages: virulent phages, where the phage infects the host bacteria, replicates and lyses the bacteria to release phage progeny; and temperate phages, which may either choose a lytic life cycle or choose to integrate into the host’s DNA and replicate alongside the host until the phage senses suitable conditions for the switch to lytic replication [16]. Prophages are temperate phages integrated into their host’s genome, and the resulting host bacteria are a ‘lysogen’. Almost every bacterial species has temperate phages, although much is still unknown about both lytic and temperate phages.

Prophages confer various benefits to their host through lysogenic conversion. The most common is superinfection exclusion: the protection of the lysogen against other phage infections [17, 18]. Many prophages also express virulence genes or toxins that promote growth of the lysogen [19–23]. Some examples of illnesses that are caused by bacteria with toxin genes provided by prophages include Shiga toxin-producing *Escherichia coli,* cholera and diphtheria.

The genetic switch that controls the decision to integrate into the host or replicate and kill the host has been at the centre of many molecular biology breakthroughs [24], such as the Nobel prizes in Physiology and Medicine in 1965 and 1969, which were awarded for discoveries regarding the viral synthesis and replication mechanisms respectively.

Many factors affect the outcome of that decision, including the concentration of bacteria, the diversity of bacterial and phage species, the redox potential of the cell (i.e. the metabolic efficiency of the bacteria), the presence of other phages, and signalling peptides that phages produce to communicate with each other [25, 26].

Here we explored the variation in prophage composition across the human body, investigated how much of the bacterial DNA in the human microbiome is provided by prophages, demonstrated how this diverges across the different areas of the human body, and quantified whether diseased microbiomes and disease-causing bacteria have different prophage abundances from the microbiomes of healthy people.

## Methods

All 949 935 publicly accessible bacterial genomes (as of 1 June 2022) listed in the dataset ‘NCBI Genome Assemblies Summary Archive 20220601’ (key resources) were downloaded from GenBank for analysis on the Flinders University HPC cluster [27]. PhiSpy [28, 29] was used to predict all of the prophages in all the bacterial genome assemblies, and we identified more than 5 million high-quality prophages [1]. PhiSpy was used as it is currently among the most accurate prophage prediction tools with the lowest runtime [30]. All the predicted prophages from all of the genomes are available from FigShare (Key Resources ‘Prophage predictions’).

We filtered the genome assemblies to exclude metagenome-assembled genomes (e.g. labelled as ‘derived from metagenome’). We also removed genomes consisting of more than 50 contigs as low quality; this cutoff ensures an average fragment length greater than 60 kb (for a typical 3 Mb genome) which was the upper length limit for almost all prophages reported in McKerral *et al*. [1]. Duplicate genome sequences were removed using the NCBI Genome Assemblies Summary (Key Resources ‘NCBI Genome Assemblies Summary Archive 20220601’). We only included genomes from humans by identifying those that had human in the host section or description. We manually sorted the remaining samples into categories and subcategories based on the area of the body from where they were isolated and the human host’s health according to metadata from Pathosystems Resource Integration Center (PATRIC) (Key Resources ‘Archive of the PATRIC Metadata from 20220601’) [31, 32].

After filtering, 20 573 unique genome accessions remained. Over half –14 987 genomes – came from bacteria associated with different human body areas. We separated those into 31 categories, with 3–2970 samples per category. Approximately half of the genomes from human-associated bacteria, 6844, could be categorized by the human host’s health. We provide these data as Key Resources ‘Prophages in humans’.

SPSS was used for statistical analysis. The Kruskal–Wallis test was used to compare the categories of genomes associated with different human body areas.

Bacterial abundances and concentrations were obtained from previous studies and are summarized in Table 1. If we found multiple different estimates, we used an average of the concentrations to compare the prophage abundances with the bacterial concentrations.

**Table 1.** Bacterial concentrations in the human body

Body site	Average no. of bacteria	Notes	Reference
Vagina	10^10^–10^11^ in total		[33]
Blood	4.19×10^7^ 16S copies ml^−1^	Only from healthy samples and low numbers (*n*=8)	[38]
Stool	0.9×10^11^	Used as a proxy for the colon	[9]
Urine	>10^3^ c.f.u. ml^−1^	Strong gender differences	[40]
Lung	10^4.5^ to 10^8.25^ copies ml^−1^		[36]
Skin	<10^11^ m^−2^		[9]
Ileum	10^8^		[9]
Breast milk	10^2^–10^4^ c.f.u. ml^−1^	Varies based on media used	[39]
Colon	10^11^ or 3.8×10^13^	Counting the total bacteria in all stool in the colon. Most of the total bacteria in the human body	[9]
Duodenum	10^3^–10^4^		[9]
Stomach	10^3^–10^4^		[9]

For the portion of genomes we could categorize by human health, we analysed multiple groups using SPSS. We compared the healthy, symptomatic and asymptomatic groups within each category with either a Kruskal–Wallis test for categories with genomes in all three groups or Mann–Whitney U-tests for the skin and gut samples that only had two variables. We combined the categories and compared healthy, symptomatic and asymptomatic groups with a Kruskal–Wallis test. Once we identified significant differences between healthy and symptomatic groups, we re-analysed the categories using only the healthy samples to determine whether the relative prophage abundances changed.

## Results

**Fig. 1.**
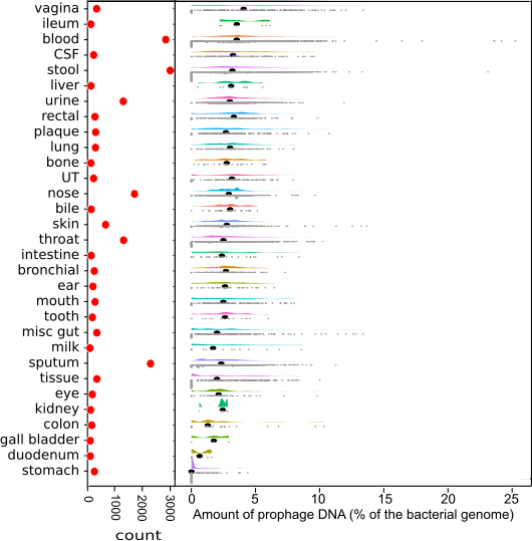
Raincloud plot of all 31 categories ordered by the mean percentage of phage DNA from highest to lowest. The red markers on the left show the number of genomes in each category while the black dots show the median of each category. CSF, cerebrospinal fluid; UT, urinary tract.

The GenBank genome assembly database contains almost 1 million publicly accessible bacterial genomes, but most are highly fragmented. However, we identified 11 513 genomes from bacteria that could be associated with different areas of the human body. These samples came from various people with different geographical locations, lifestyles, ages, diets and medical conditions. We identified prophages in these genomes and calculated the percentage of the prophage sequence genomes for each sample source location (Fig. 1).

There is large variation in the proportion of prophage DNA within and between different body sites. The average prophage DNA content ranges from 0 to 5 % of the bacterial genome. While many areas have a average prophage DNA content closer to 2–3 %, there is a sizeable difference between body sites, especially at the extremes – vagina and blood at the high end, with 4–5 % prophage content, and duodenum and stomach at the lower end, with close to 0 % prophage content.

The vaginal samples had the highest average proportion of prophage DNA. A single genus of bacteria dominates the healthy vaginal microbiome *– Lactobacillus* – which produces antimicrobial compounds that control other bacterial populations [33]. The vaginal microbiome is also dense, containing 10^10^–10^11^ bacterial cells [33]. Both high bacterial concentrations and a microbiome dominated by a few species are two factors previously shown to correlate with higher rates of lysogeny [34].

Conversely, the stomach had the lowest average proportion of prophage DNA. No prophages could be detected in most (76.67%) of the genomes from bacteria isolated in the stomach. The stomach is significantly different from almost every other body site and is one of the most extreme environments in the human body. A handful of genera dominate, and the bacterial concentrations are relatively low, in the order of 10^3^–10^4^ bacteria [35]. Overall, it is quite the opposite of the vaginal microbiome.

### Respiratory and gastrointestinal tracts

**Fig. 2.**
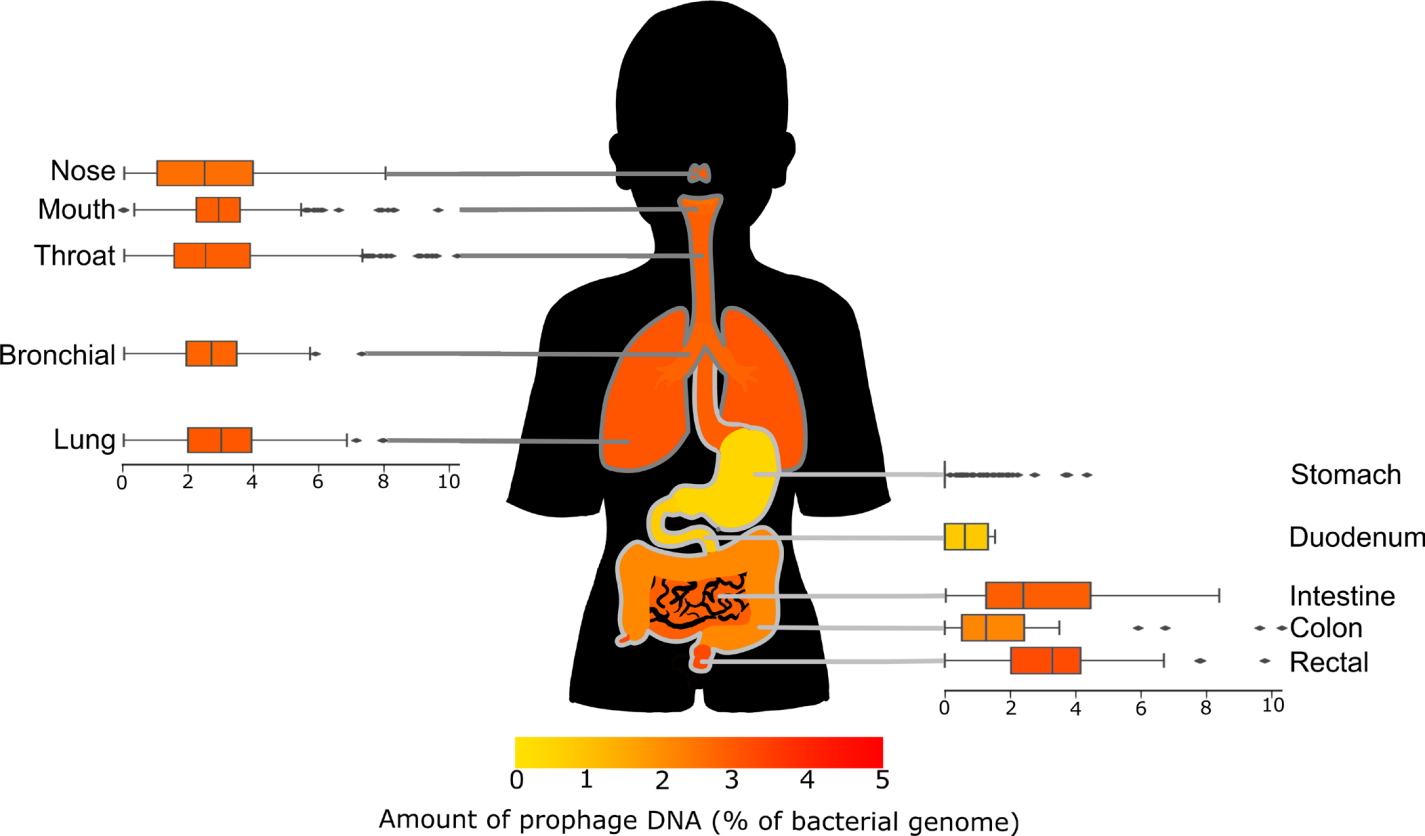
Box plots showing the amount of prophage DNA in each area of the respiratory and gastrointestinal tracts. The figure is coloured by the average proportion of prophage DNA as displayed in the scale below.

Narrowing our focus to the respiratory and gastrointestinal (GI) tracts allows us to examine how the microbiome changes as the conditions change in transit from the mouth to anus. We juxtaposed the distributions of prophage DNA with the GI and respiratory systems for visual assessment (Fig. 2), and we performed Kruskal–Wallis statistical tests to determine if these distributions were significantly different.

The different sections of the respiratory tract have similar distributions of prophage DNA, reflecting similar conditions. The areas also connected to the GI tract – the mouth and throat – were significantly different to the lungs (*P*<0.05), while the nose was only significantly different to the throat (*P*<0.005).

The lungs had the highest prophage DNA, while the mouth had the lowest. The microbiome of the respiratory tract changes with the age of the host, becoming more diverse as the human matures from infant to adult [36–38]. Since the abundance of temperate phages correlates with microbial diversity, there may be fewer temperate phages in the respiratory tract of older people. The overall bacterial concentration estimates suggest the mouth has more bacteria than the lungs [9, 36], which is generally conducive to higher rates of lysogeny. However, we observe the opposite trend with lung-isolated genomes having higher proportions of prophage DNA.

Conversely, the GI tract has a much wider range of bacterial concentrations and does not follow a linear order like the respiratory tract. The distinct areas of the GI tract have much more varied environments, and the prophages appear to follow the piggyback-the-winner model, with more prophages in areas of the body with higher bacterial concentrations, such as the stool or mouth. In contrast, the more hostile environments such as the stomach have less prophage DNA per bacteria.

Overall, bacterial concentration alone does not adequately explain the proportion of prophage DNA, and we must look to other factors to explain our results. Generally, bodily fluid samples (e.g. breast milk, urine and blood) had lower bacterial concentrations [37–40] but higher prophage concentrations than the other body sites.

### Effects of host health

**Fig. 3.**
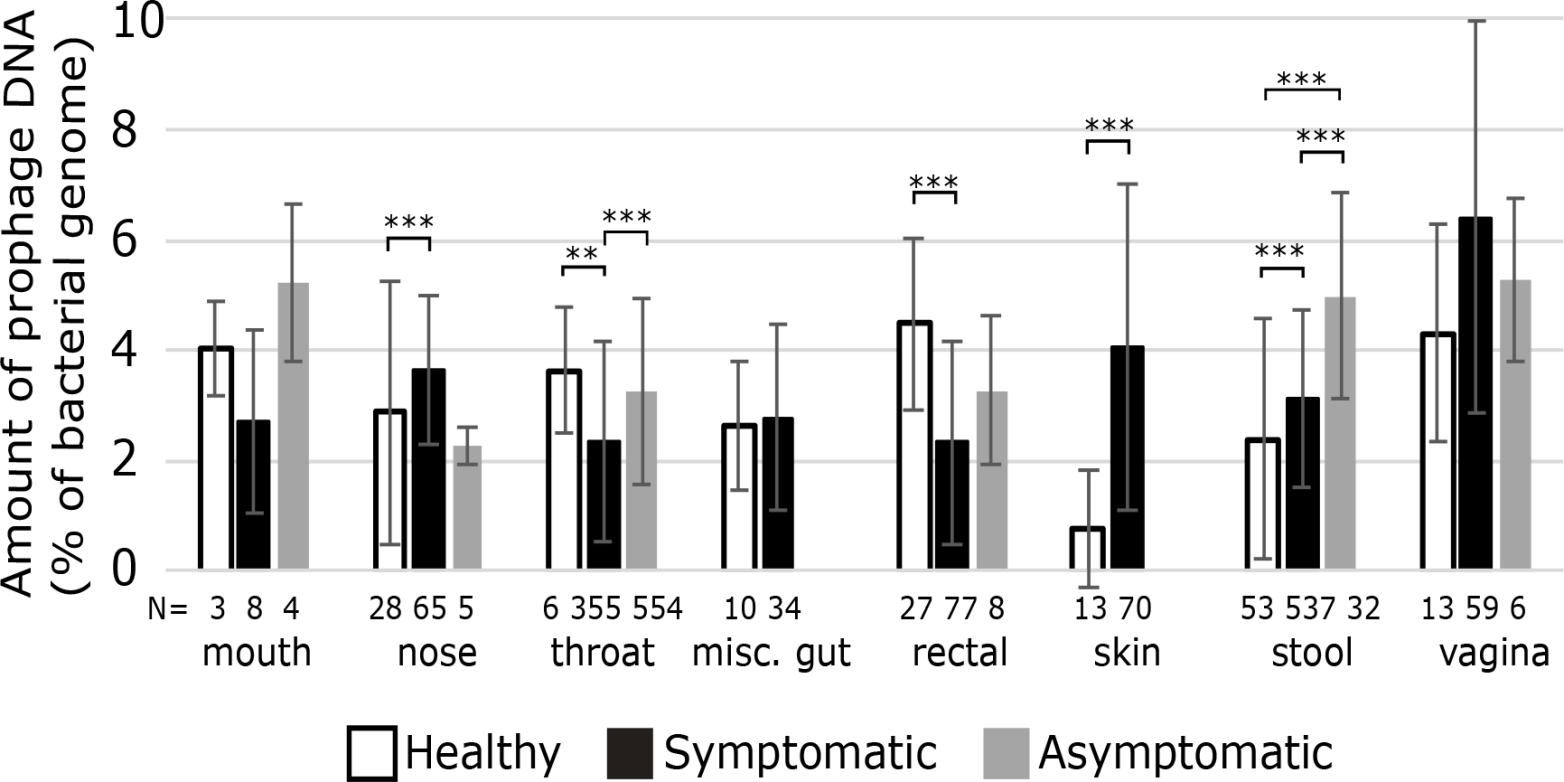
Each area of the human body with at least three genomes and described as sampled from healthy people. The number beneath each column indicates the number of samples in each group. Error bars represent 1 sd, while asterisks represent significant differences (**P<0.005, ***P<0.001).

Many of the samples were clinical samples which probably influenced the results, for instance with bacteria that dominate in dysbiotic microbiomes and specific disease-causing bacteria. To examine if these clinical samples exhibit different lysogenic profiles, we searched the metadata for genomes that had data on their host’s health and split the samples into groups based on whether the sample metadata listed the human as healthy, having various ailments (including diseases caused by specific bacteria and other ailments involving various bacteria/viruses) or asymptomatic. We independently assessed the samples by body site when investigating differences in the number of prophages per genome and determined significant differences using a Kruskal–Wallis test (Fig. 3).

Only eight body sites had samples from healthy individuals (Fig. 3). The nose, skin and stool samples all had more prophages in samples from symptomatic patients than healthy individuals, suggesting that prophages may contribute to disease at these sites. In contrast, the throat and rectal samples had fewer prophages in symptomatic individuals than in healthy people.

Both throat and stool samples had significantly more prophages in asymptomatic individuals than in healthy individuals. Typically, patients are classified as asymptomatic when they have a disease but are not currently experiencing symptoms. The difference in prophage abundance could suggest that prophages are either decreasing the virulence of their hosts in these areas or providing greater survivability so that once the illness clears, predominantly lysogens remain.

Related samples, such as from the lower GI tract (stool and rectal) or the respiratory system (nose, mouth and throat), did not always show similar patterns of prophage abundance. The bacterial species, types of illnesses or the different types of tests used at different sites could eliminate patterns between body sites.

### Effects of host region

**Fig. 4.**
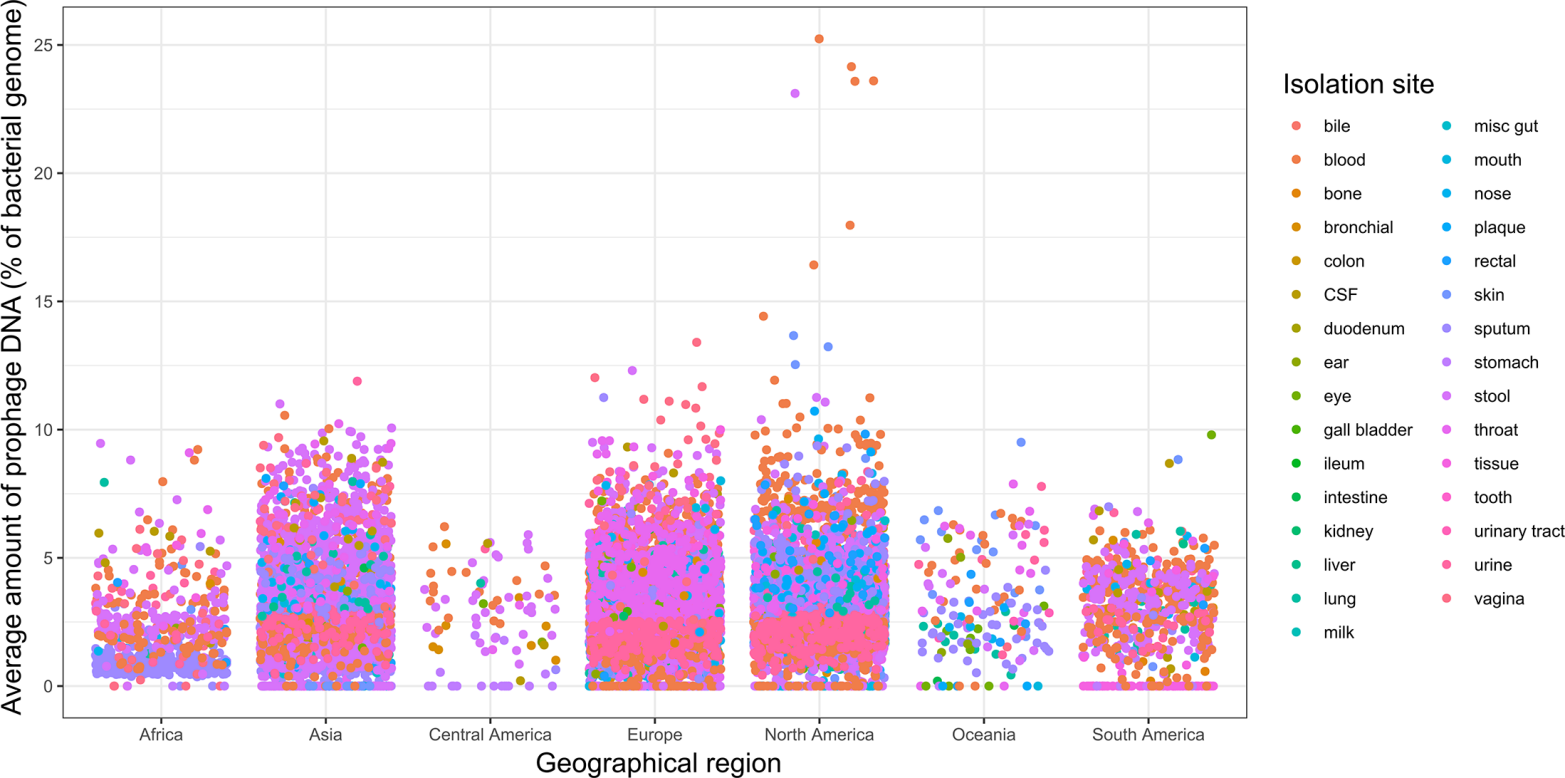
Jitter plot grouping samples by geographical region. Area of the body is represented by dot colour. CSF, cerebrospinal fluid.

One factor known to affect the microbiome is geography. We performed a Kruskal–Wallis test to compare prophage abundance by geographical region. We found that African samples had significantly fewer prophages than all of the other regions. South and Central America were significantly different from Asia and North America. North America was also significantly different from Europe and Oceania was only significantly different from Africa (Fig. 4).

**Fig. 5.**
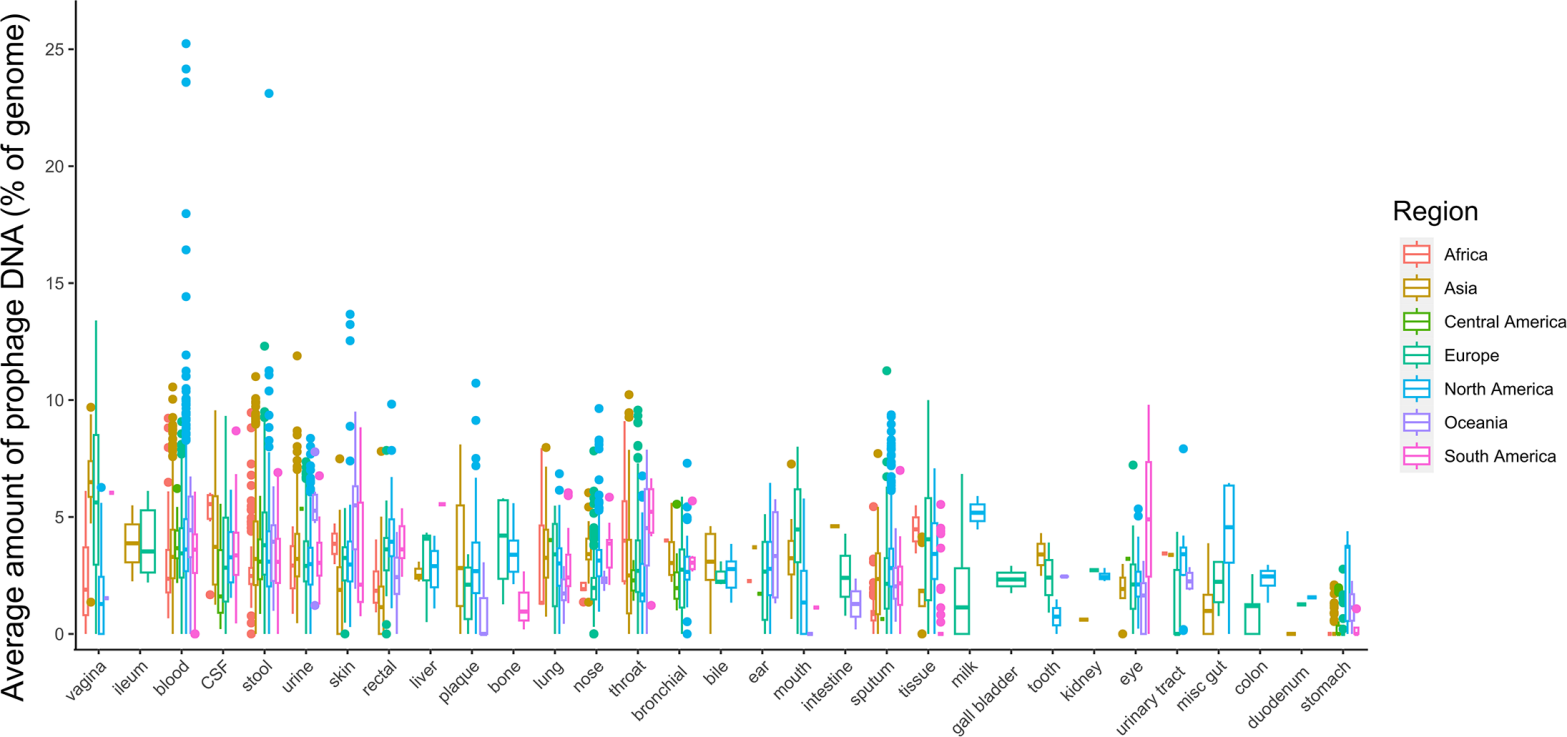
Box plots for each area of the body split by isolation region. Box colour represents geographical region. CSF, cerebrospinal fluid.

We further applied Kruskal–Wallis tests to compare both area of the body and geographical region. We show that not every area of the body differs according to geographical location, and, most interestingly, the gut-derived genomes did not vary significantly between North America and Europe, whereas the oral samples showed a significant difference (Fig. 5).

## Discussion

Different areas of the human body had different numbers of bacterial genomes associated with them (Fig. 1; red markers), and almost every area of the body had considerable variation in the number of prophages. This variation could be due primarily to the differences in microbiome bacterial compositions between the individuals sampled as prophages are very host-specific and different species and even different strains of bacteria are the hosts of different phages. While there is evidence for a ‘core microbiome’ of functional genes [41–43], the taxonomic makeup of the microbes between individuals varies significantly [44]. Many factors affect the composition of the human microbiome, including diet, medications, overall health and fitness, and weight [2, 5, 15, 41, 45, 46]. Different areas of the human body are affected by some factors more that others. The gut microbiome, in particular, is affected by geographical variations [47, 48] and host demographics [49], with *Firmicutes* increasing with latitude and *Bacteroides* decreasing regardless of host ethnicity [47].

Some of these bacteria enter the hunam body through the mouth and nose as we eat, drink and breathe, and some of these bacteria find their way down further into the respiratory or GI systems to supplement the human microbiome [36]. This results in the connected microbiomes, such as of the mouth and throat, having similar compositions, and our results showed that while the prophage concentrations often followed a similar pattern, there were a few outliers. For example, the stomach differed from the rest of the GI tract in every way. This could be due to different bacteria being present or different bacteria becoming dominant in the microbiome. There are two main hypotheses regarding lysogeny rates: piggyback-the-winner and piggyback-the-persistent. Piggyback-the-winner suggests that the microbiomes with high bacterial concentrations are more likely to favour lysogeny [3], while the piggyback-the-persistent suggests the opposite [50]. The lungs have a lower bacterial concentration, yet a relatively high amount of prophage DNA suggests that it follows the persistent strategy of piggyback-the-persistent, while there were less apparent patterns in the gut.

Because different bacteria have different prophages and lower bacterial diversity is associated with higher rates of lysogeny [5, 45, 49], external factors that affect the makeup of the human microbiome could affect the number of prophages in each bacterial genome. Human health is perhaps the most critical factor that influences our microbiomes. Illnesses and generally poorer health are often associated with less diverse microbiomes, particularly in the respiratory and GI systems [5, 45, 51]. This would suggest that bacteria associated with disease should have more prophages on average than bacteria associated with healthy microbiomes; however, we found wide variation in the prophages between healthy and symptomatic samples in different body sites, with some areas of the body having significantly more prophage DNA per bacteria in healthy samples, suggesting that not all disease-causing bacteria have more prophages than those in the healthy microbiome. There were some differences in the averages of body site between healthy samples and all samples, but most samples lacked metadata on host health so this factor could not be widely applied to most areas of the body.

The last factor we were able to test was the isolation country. The gut microbiome is affected by location [47–49], and it is reasonable to assume that prophage abundance may also be affected. While our dataset only includes gut samples for three regions – Asia, North America and Europe – we were able to show that North America and Europe were significantly different (Fig. 5). Overall, our findings suggest that the human microbiome varies by location, with samples taken from Africa being significantly different from the rest of the world, while regions such as Europe and Asia were more similar. This remained true for most areas of the human body, with a few notable exceptions. This could potentially be due to rates of movement between regions. There is wide variation in the amount of prophage DNA in the bacterial genomes of the human microbiome. Categorizing the samples by body site revealed patterns in prophage abundance. Areas connected or with similar environments often had a similar prophage distribution. The respiratory tract, which has a lower microbial load, appears to follow the piggyback-the-persistent scenario, while the microbially rich GI tract follows the piggyback-the-winner scenario. The microbiome impacts human health, and vice versa, and a few body sites showed significant differences in prophage abundance in health and disease.
